# Silicon in Plants: Alleviation of Metal(loid) Toxicity and Consequential Perspectives for Phytoremediation

**DOI:** 10.3390/plants12132407

**Published:** 2023-06-21

**Authors:** Daniel Puppe, Danuta Kaczorek, Mathias Stein, Jörg Schaller

**Affiliations:** 1 Leibniz Centre for Agricultural Landscape Research (ZALF), 15374 Müncheberg, Germany; 2 Department of Soil Environment Sciences, Warsaw University of Life Sciences (SGGW), 02-776 Warsaw, Poland

**Keywords:** heavy metals, phytoliths, complexation, co-precipitation, abiotic stress, metal(loid) sequestration

## Abstract

For the majority of higher plants, silicon (Si) is considered a beneficial element because of the various favorable effects of Si accumulation in plants that have been revealed, including the alleviation of metal(loid) toxicity. The accumulation of non-degradable metal(loid)s in the environment strongly increased in the last decades by intensified industrial and agricultural production with negative consequences for the environment and human health. Phytoremediation, i.e., the use of plants to extract and remove elemental pollutants from contaminated soils, has been commonly used for the restoration of metal(loid)-contaminated sites. In our viewpoint article, we briefly summarize the current knowledge of Si-mediated alleviation of metal(loid) toxicity in plants and the potential role of Si in the phytoremediation of soils contaminated with metal(loid)s. In this context, a special focus is on metal(loid) accumulation in (soil) phytoliths, i.e., relatively stable silica structures formed in plants. The accumulation of metal(loid)s in phytoliths might offer a promising pathway for the long-term sequestration of metal(loid)s in soils. As specific phytoliths might also represent an important carbon sink in soils, phytoliths might be a silver bullet in the mitigation of global change. Thus, the time is now to combine Si/phytolith and phytoremediation research. This will help us to merge the positive effects of Si accumulation in plants with the advantages of phytoremediation, which represents an economically feasible and environmentally friendly way to restore metal(loid)-contaminated sites.

## 1. Introduction

Numerous prokaryotes as well as eukaryotes have been evolutionarily adapted to use dissolved silicon (Si) in the form of monomeric silicic acid (H_4_SiO_4_) for the formation of hydrated amorphous silica (SiO_2_ ∙ *n*H_2_O) in a process called biosilicification [1]. Based on their origin, biogenic silica (BSi) structures and residues in soils represent BSi pools that can be distinguished as follows: (i) bacterial BSi (formed in bacteria), (ii) fungal BSi (formed in fungi), (iii) phytogenic BSi (formed in plants), (iv) zoogenic BSi (formed in animals), and (v) protistic BSi (formed in protists) [2].

Phytogenic silica can be found (i) in living plants within cells (i.e., in the cell wall and the cell lumen) forming relatively stable, recognizable phytoliths, that can be found in soils as plant microfossils or (ii) in intercellular spaces and extracellular (cuticular) layers forming relatively fragile silica structures [3,4]. Phytoliths are mainly made of SiO_2_ · *n*H_2_O, but also commonly contain organic matter and various elements like aluminum (Al), calcium (Ca), iron (Fe), manganese (Mn), and phosphorus (P) [5,6]. Against this background, the potential of carbon sequestration in phytoliths is under controversial discussion recently [7]. For the majority of higher plants, Si is considered a beneficial substance nowadays because of the various favorable effects of Si accumulation in plants that have been revealed, i.e., increased plant growth and resistance against biotic and abiotic stresses like fungal infections, drought, or heavy metal toxicity [8,9,10].

The term “heavy metals” often refers, but is not limited, to chromium (Cr), cobalt (Co), nickel (Ni), copper (Cu), zinc (Zn), arsenic (As), cadmium (Cd), tin (Sn), mercury (Hg), and lead (Pb). As the term “heavy metal” is misleading and imprecise, because it is not clearly defined and often mixes metals and metalloids that are associated with environmental contamination and potential toxicity, we use the term “metal(loid)” instead [11]. Based on their role in organisms, metal(loid)s are categorized as essential (e.g., Cu, Fe, Mn, Ni, or Zn) or non-essential (e.g., Pb, Cd, As, or Hg), whereby essential metal(loid)s can also become toxic when they exceed specific concentrations. The accumulation of non-degradable metal(loid)s in the environment strongly increased in the last decades by intensified industrial and agricultural production with negative consequences for the environment and human health [12,13,14].

Phytoremediation is referred to all methods that use plants to (i) extract and remove elemental pollutants from contaminated soils or (ii) to decrease the bioavailability of these pollutants in soils [15]. In comparison to many other soil remediation methods, which are typically expensive, disruptive, and appropriate only for small areas, phytoremediation represents a cost-effective and environmentally friendly alternative. Moreover, plants’ roots are able to absorb metal(loid)s that are present in soils at concentrations too low for physicochemical remediation techniques. This is why phytoremediation has been commonly used for the restoration of contaminated sites, e.g., former surface mining areas [16,17,18,19].

However, it should be noted that phytoremediation is frequently slower than conventional engineering technologies, and thus has to be considered as a long-term remediation solution. In fact, decreasing metal(loid) concentrations of contaminated soils to environmentally safe levels and below specific regulatory limits might take decades to several hundreds of years [20,21]. Factors that mainly influence phytoremediation duration are contaminant concentrations in soils, size of the contaminated area, and plant-specific properties (e.g., growing time, biomass, and the potential to accumulate metal(loid)s). In this context, hyperaccumulators, i.e., plant species that can safely accumulate metal(loid)s in concentrations 100- to 10,000-times higher than in non-hyperaccumulating species [22,23,24] with high biomasses might be most qualified for a fast remediation of contaminated sites.

In general, the potential of Si for phytoremediation has not been addressed in the literature until now, although the knowledge of the benefits of Si for the alleviation of metal(loid) toxicity in plants has been well known for decades. Thus, in our viewpoint article, we aim to join the current knowledge of beneficial Si accumulation effects in plants to the knowledge of phytoremediation advantages, i.e., economic efficiency and environmental friendliness. For this purpose, we briefly summarize the current knowledge of Si-mediated alleviation of metal(loid) toxicity in plants and the potential role of Si in the phytoremediation of soils contaminated with metal(loid)s. In this context, a special focus is on metal(loid) accumulation in (soil) phytoliths. Our summary ends with concluding remarks, where future directions are outlined. As sustainability and environmental compatibility have become more topical than ever, we hope that our article will help foster future research on the promising role of plant Si in metal(loid) toxicity alleviation and the phytoremediation of metal(loid)-contaminated soils.

## 2. Silicon Uptake and Accumulation in Plants

Although Si is very abundant in Earth’s crust (>90 vol. % consist of SiO_2_ and silicates), Si bioavailability is often limited in soils because plant-available Si (H_4_SiO_4_) is (i) leached as a result of rainfall and irrigation, especially in agricultural soils; (ii) is bound to the surface of minerals and their competition for binding sites with, e.g., phosphorus or organic carbon; and (iii) is subject to polymerization/precipitation reactions [25]. Soils with a low Si bioavailability generally can be characterized as highly weathered, leached, acidic, and low in base saturation.

Si contents vary considerably between plant species with values ranging from about 0.1 to 10% Si per dry mass [26]. Based on their Si content, plants have been traditionally divided into three groups, i.e., (i) non-accumulators or excluders (a Si content per dry mass < 0.5%), (ii) intermediate accumulators (a Si content per dry mass of 0.5–1%), and (iii) accumulators (a Si content per dry mass > 1%) [27]. Field crops, especially cereal grasses of the family Poaceae (or Gramineae), are known as Si accumulators. Si absorption by plants is controlled by two different types of Si transporters (called “Low silicon”, Lsi), i.e., specific influx (called Lsi1 and Lsi6) and efflux (called Lsi2 and Lsi3) channels, which have been found especially in crops like rice (*Oryza sativa*), wheat (*Triticum aestivum*), or sorghum (*Sorghum bicolor*) [28,29]. While Lsi1 and Lsi6 represent aquaporins, which allow for the passive diffusion of H_4_SiO_4_ across the plasma membrane, Lsi2 and Lsi3 are proton (H^+^) antiporters that can export H_4_SiO_4_ from cells. However, it should be kept in mind that the mechanisms behind the uptake, transport, and accumulation of Si in plants (active vs. passive Si transport) as well as Si-induced plant resistance (mode of action of Si in plants) are still not fully understood, and thus are under controversial discussion [30,31,32,33].

The size of Si precipitates in plants ranges from about 100 nm to 1 mm [34,35], whereby phytogenic Si can be found in almost all plant organs, e.g., in leaves, stems, and roots [36]. In this context, the mode of silica deposition in plants seems to be organ-specific. In roots, e.g., three basic modes of Si deposition have been identified, i.e., (i) impregnation of endodermal cell walls (e.g., in wheat), (ii) formation of Si aggregates associated with endodermal cell walls (e.g., in sorghum and sugarcane), and (iii) formation of Si aggregates in “stegmata” cells forming a sheath around sclerenchyma fibers (e.g., in some palm species) [37]. On the contrary, in stems and leaves, silica is mainly deposited in the epidermis [38]. For plant microfossils (soil phytoliths), an international nomenclature based on phytolith morphology has been developed, which is especially used in archeological, paleo-environmental, evolutionary, taxonomic, and climatological studies for the taxonomic identifications of plants [39].

Si uptake and storage in plants have been analyzed for several ecosystems. Regarding natural ecosystems, Si storage in aboveground vegetation has been reported, e.g., for the Great Plains [40], the tropical humid grass savanna [41], or forested biogeosystems [42,43,44]. Si uptake at agricultural sites has been reported for, e.g., wheat, rice, and sugarcane, which represent Si accumulators with relatively high Si contents as well as biomasses [45,46,47]. In contrast to natural ecosystems, where BSi is recycled to great amounts, agricultural sites are subject to high Si exports by harvest, gradually depleting plant-available Si in soils (anthropogenic desilication) year by year [48,49]. To ensure a sufficient Si supply for plants, soil and foliar Si fertilizers are widely used, especially in rice and sugarcane production [10,50]. In this context, Si-rich sludges or slags are frequently used as Si sources as they show appropriate characteristics like high Si solubility and a reasonable cost/benefit ratio. However, due to the fact that these materials are often metal(loid)-laden, a potential metal(loid) contamination of the food chain has to be carefully evaluated [51]. In contrast, crop straw recycling has been identified as a promising, environmentally friendly alternative for increasing Si bioavailability in agricultural soils, and thus also a promising alternative for preventing anthropogenic desilication in the long term [52].

## 3. Silicon-Mediated Metal(loid) Toxicity Alleviation in Plants

In general, there are numerous publications on various mechanisms of the Si-mediated alleviation of metal(loid) toxicity in plants, comprising processes in soil (solution) and plants [53,54].

In soils, metal(loid)s are complexed with dissolved silicic acid, forming slightly soluble metal–silicate complexes, especially if metal and Si concentrations and soil pH are sufficiently high [55,56,57]. More recently, it was shown that particulate compounds formed from the reaction between silicic acid and metals at concentrations restrict the precipitation of metal silicates in aqueous solution [58]. In this context, the metals, particularly Cu, were structurally incorporated into the polymeric network of polymerizing silicic acid. Moreover, those compounds might also form in acidic soils, removing metals from solution by the same processes, e.g., adsorption on surface silanol groups, diffusion/occlusion in the interior of polymerizing silica, and structural incorporation, depending on the metal [59].

The main mechanisms in planta potentially comprise (i) complexation and co-precipitation of metal(loid)s with Si, (ii) Si-induced stimulation of antioxidant systems, (iii) Si-mediated enhanced photosynthesis efficiency, and (iv) Si-induced alterations in membrane transport-related gene expression.

Complexation and co-precipitation of metal(loid)s with Si in metabolically less active cell compartments like cell walls might inhibit the allocation of toxic metal(loid)s within other plant tissues that play important roles in plant metabolism [60,61,62]. Some studies of Si foliar fertilization, e.g., showed reduced metal(loid) concentrations and accumulation in plant parts, which are of agricultural interest, e.g., rice grains [61,63,64]. As evaporation plays an important role in silica precipitation, modifications of transpiration rates of plants by Si supply can affect silica deposition (silicification) [65]. In this context, silicification in plants seems to be initiated and controlled by specific cell wall polymers and proteins [33].

Stimulation of antioxidant systems in plants seems to play a further important role in alleviating metal(loid) toxicity [66,67,68,69]. Generally, Si can reduce oxidative stress induced by metal(loid) toxicity by enhancing the activities of enzymatic (e.g., superoxide dismutase, peroxidase, and catalase) and non-enzymatic (e.g., ascorbic acid and glutathione) antioxidants. This decreases the accumulation of reactive oxygen species like hydrogen peroxide (H_2_O_2_) and hydroxyl radicals (•OH), which are responsible for errors in cell signaling pathways that cause severe cell damage or death [70,71,72]. Cooke and Leishman [73] statistically assessed the responses of plants under abiotic stress to Si application in a meta-analysis. They found that Si consistently alleviates oxidative stress and that responses differed among plant families.

Enhanced photosynthesis efficiency seems to result from different effects induced by Si fertilization like metal(loid)–Si complexation and co-precipitation and the stimulation of antioxidant plant systems. In consequence, a reduction in harmful effects on the photosynthetic apparatus and enhanced chlorophyll biosynthesis under metal(loid) stress can be observed [74,75,76]. These findings are corroborated by the meta-analysis of Cooke and Leishman [73], who found that Si addition significantly increased photosynthetic rates and total chlorophyll concentrations in plants under abiotic stress. In general, the mode of action of Si fertilization on photosynthesis under metal(loid) stress is not clarified in detail yet and both direct (i.e., an active Si influence on photosynthesis) and indirect (i.e., photosynthesis profits from other beneficial plant impacts) effects of Si application are discussed [53,77]. In this context, Nwugo and Huerta [78] identified 50 proteins associated with, e.g., photosynthesis and pathogen response that were significantly regulated by Si, indicating an active involvement of Si in plant physiological processes.

Si-induced alterations in membrane transport-related gene expression might also play a role in the alleviation of metal(loid) toxicity in plants. Studies with Si-supplied *Arabidopsis thaliana* and rice plants showed up- and down-regulations of Si and metal transport-related gene expressions, respectively [79,80,81]. However, as our current knowledge of Si accumulation in plants on the molecular level is still in its infancy, further research is necessary to unravel the underlying mechanisms. In fact, only few Si influx and efflux channels (namely Lsi1, Lsi2, Lsi3, and Lsi6) have been identified in a limited number of plant species like rice, barley (*Hordeum vulgare*), wheat, maize (*Zea mays*), cucumber (*Cucumis sativus*), pumpkin (*Cucurbita moschata*), and soybean (*Glycine max*) [28].

## 4. Metal(loid) Accumulation in Phytoliths

While knowledge of enhanced metal(loid) tolerance in plants induced by Si dates back to at least the 1950s (see, e.g., Ma and Takahashi [27] and the references therein), the accumulation of metal(loid)s within phytogenic silica or phytoliths is a phenomenon that has been reported as of the end of the 1990s or the beginning of the 2010s, respectively (Table 1). Neumann et al. [82], e.g., found Zn-silicates in the epidermal cell walls of *Minuartia verna* and Bringezu et al. [83] described the accumulation of Zn and Sn within silicates in the cell walls of *Silene vulgaris*.

Buján [84] analyzed the elemental composition of phytoliths from different species of the plant family Ericaceae and found numerous metal(loid)s entrapped in these phytoliths. Kameník et al. [5] analyzed barley phytoliths and showed (i) that these phytoliths were enriched in terrigenous elements (e.g., Al or Fe), but depleted in the elements that represent the major inorganic constituents of plants (e.g., potassium (K) or Ca), and (ii) that phytoliths originating from various plant parts differ in elemental composition. Nguyen et al. [85] and Tran et al. [86] reported the encapsulation of Pb and Cu in rice and grass phytoliths, respectively. Some more studies of the accumulation of metal(loid)s in phytoliths of different plants followed recently [87,88,89,90] (details can be found in Table 1). Most recently, Liu et al. [91] analyzed the encapsulation of toxic trace metal(loid)s in wheat phytoliths. They found that As and Cr were more often encapsulated in wheat phytoliths than Cd, Pb, Zn and Cu, which were mainly accumulated in organic tissues, demonstrating that the potential interaction of plant silica with metal(loid)s is highly variable among elements.

In this context, the origin of phytoliths (cell wall vs. cell lumen phytoliths) might also play an important role in metal(loid) accumulation. While cell wall phytoliths are associated with a carbohydrate matrix, lumen phytoliths seem to contain more proteins and glycoproteins than cell wall phytoliths, which has consequences for phytolith dissolution kinetics and carbon sequestration [3,7]. If metal(loid)s are accumulated to different amounts in cell wall and lumen phytoliths, the ratio between cell wall and lumen phytoliths in a specific plant will be of great interest for the storage of metal(loid)s in phytoliths. Grasses and cereals, e.g., seem to contain more lumen phytoliths compared to other plant groups [92]. However, as there is no information on metal(loid) accumulation in different types (i.e., cell wall vs. lumen phytoliths) of phytoliths yet, future research on this aspect is urgently needed (see Section 6).

Moreover, analyses of metal(loid) accumulation in phytoliths have been largely limited to the plant family Poaceae, which is known for its Si-accumulating plant species (Table 1). In this context, mainly phytoliths from aboveground plant materials were analyzed. Thus, there is no or only little information on metal(loid) accumulation in root phytoliths or phytoliths extracted from soils, respectively. In fact, root phytoliths might be another important location for the accumulation of metal(loid)s as they can be abundantly found in some plants, e.g., grasses [93,94,95]. Soil phytoliths originating from the litterfall of metal(loid)-accumulating plants might represent an important sink for metal(loid)s in soils [85,86]. However, more studies are needed to better understand this potential pathway of metal(loid) (long-term) sequestration in soils (see Section 6).

## 5. Consequential Perspectives for Phytoremediation

In general, remediation strategies that are using plants can be divided in five main subgroups, i.e., (i) phytodegradation (breakdown of pollutants by plant enzymes), (ii) phytoextraction (accumulation of pollutants in harvestable plant tissues), (iii) phytostabilization (reduction in the mobility and bioavailability of pollutants in the environment by plants), (iv) phytovolatilization (transformation of harmful elements into less dangerous ones within the plant and subsequent release in volatile form via leaves), and (v) rhizofiltration (filtering polluted water by plant roots) [19,96,97,98].

As Si generally enhances plant performance in various ways [99], all of these phytoremediation strategies might benefit from a Si supply. However, below we focus on the remediation strategies that are suitable to mitigate the metal(loid) contamination of soils, i.e., phytoextraction, phytostabilization, and phytovolatilization (phytodegradation and rhizofiltration are excluded because these strategies are limited to organic pollutants and aquatic environments, respectively).

**Figure 1:**
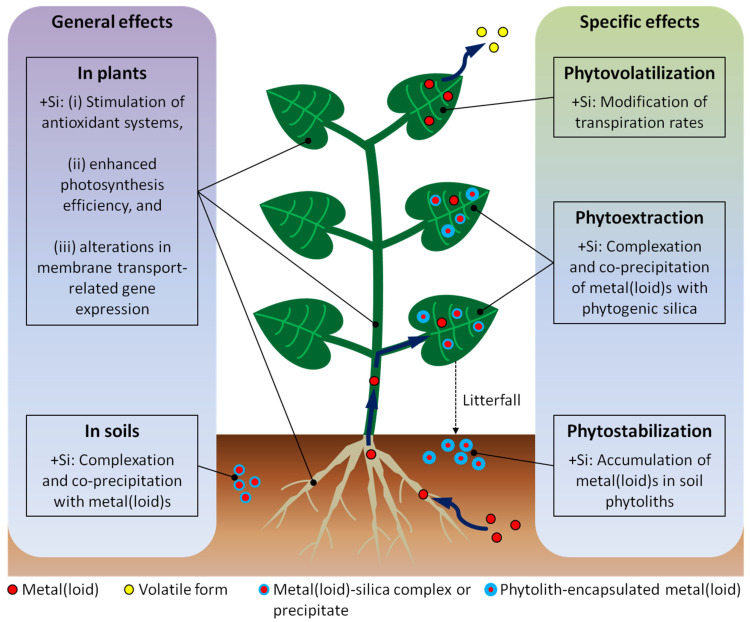
Effects of Si supply (+Si) on metal(loid) toxicity alleviation in plants and soils (general effects, left) and implications for the discussed phytoremediation techniques (specific effects, right). Metal(loid)s, their volatile forms, metal(loid)-silica complexes or precipitates, and phytolith-encapsulated metal(loid)s are indicated by different symbols (see key along the bottom).

Phytoextraction might benefit from a Si supply by the enhanced complexation and co-precipitation of metal(loid)s with phytogenic silica (see Table 1). In this context, the accumulation of metal(loid)s in soil phytoliths might be a promising pathway for the long-term sequestration of metal(loid)s in soils, and thus for the phytostabilization of these elements. Phytovolatilization, which is most effective in climates with low relative humidity and high evapotranspiration, might benefit from modifications of plant transpiration rates by Si supply. Aside from these specific effects, phytoextraction, phytostabilization, and phytovolatilization might generally benefit from a Si supply of plants by a Si-induced reduction in oxidative stress caused by metal(loid) uptake, a Si-mediated enhanced photosynthesis efficiency, and Si-induced alterations in membrane transport-related gene expression (Figure 1).

Although the knowledge of the benefits of Si for the alleviation of metal(loid) toxicity in plants has been well known for decades (see Section 3), studies on phytoremediation have mostly not addressed this aspect. In this connection, elemental analyses in phytoremediation studies were mainly limited to the metal(loid)s of interest (i.e., the pollutant), but not to Si. Thus, for many metal(loid)-accumulating plants, specific Si contents are just unknown (Table 2). However, some plant families that include known metal(loid) accumulating plants (Table 2), are also known for Si accumulating plant species, e.g., the families Poaceae, Cyperaceae, or Fagaceae [26]. While metal(loid) accumulation in phytoliths of plants of the Poaceae family has attained some scientific attention recently (see Table 1), we have almost no information on this aspect regarding other plant families. Thus, the time is now to combine Si/phytolith and phytoremediation research (see Section 6). This will help us join the positive effects of Si accumulation in plants to the advantages of phytoremediation, which represents an economically feasible (relatively low cost of installation and maintenance) and environmentally friendly way to restore metal(loid)-contaminated sites [100,101].

## 6. Conclusions and Future Directions

Against the background of global change with increased industrial production and a growing global population, there is an ample need for sustainable and environmentally friendly strategies to mitigate human-caused environmental pollution. In this context, Si-enhanced phytoremediation methods seem to represent the means of choice, if the factor of time plays a minor part. Moreover, the accumulation of metal(loid)s in phytoliths might offer a promising pathway for the long-term sequestration of metal(loid)s in soils. However, to better understand the role of Si in phytoremediation and to evaluate the potential of metal(loid) storage in the soils’ phytolith pool, the following questions have to be resolved in future studies.

(i) Which plants are particularly suitable for the accumulation of metal(loid)s in phytoliths? In this context, grasses (Poaceae) seem to be very promising candidates, as they usually show relatively high Si contents as well as biomasses [40,103,104]. However, herbs, shrubs, or fast-growing trees should also be considered in future studies [105,106]. Finding plant species that hyperaccumulate metal(loid)s and that show relatively high Si contents as well as biomasses might accelerate the phytoremediation duration for a specific site considerably.

(ii) Which metal(loid)s are accumulated in phytoliths and are there differences between cell lumen and cell wall phytoliths? If yes, the ratio of cell lumen to cell wall phytoliths [107] might be a good indicator for the evaluation of the suitability of a specific plant species for metal(loid) accumulation in phytoliths.

(iii) Can cereal crops be used for phytoremediation of metal(loid)-contaminated agricultural soils? In this context, it should be ensured that metal(loid)s are mainly accumulated in the non-edible parts of the plants and that specific metal(loid) concentrations in the fruits are non-hazardous to health [108].

(iv) How long can metal(loid)s be stored in different (cell lumen and cell wall) phytoliths in soils? As phytolith dissolution is largely pH dependent, the effects of agricultural practices (e.g., liming) on metal(loid) release from phytoliths have to be analyzed in detail [85,86].

The answers to these questions will help us (i) evaluate the potential of specific plants for the phytoremediation of metal(loid)-contaminated (agricultural) sites, (ii) better understand metal(loid) accumulation in different phytoliths, and finally (iii) identify phytoliths that are suitable for the long-term sequestration of metal(loid)s in soils.

## Figures and Tables

**Table 1:** Accumulation of metal(loid)s in phytogenic silica or phytoliths.

Year	Metal(loid)	Plant	Location of Metal(loid) Accumulation	Reference
1997	Zinc (Zn)	*Minuartia verna* (Caryophyllaceae)	Silicates in epidermal cell walls of leaves	Neumann et al. [82]
1999	Zinc (Zn), Tin (Sn)	*Silene vulgaris* (Caryophyllaceae)	Silicates in epidermal cell walls of leaves	Bringezu et al. [83]
2013	Diverse	Different species (Ericaceae)	Phytoliths in leaves	Buján [84]
2013	Diverse	*Hordeum vulgare* (Poaceae)	Phytoliths in awns, stems, and leaves	Kameník et al. [5]
2019	Lead (Pb)	*Oryza sativa* (Poaceae)	Phytoliths in rice straw and soils	Nguyen et al. [85]
2019	Copper (Cu)	Grasses dominated by *Axonopus compressus* (Poaceae)	Phytoliths in grass shoots and soils	Tran et al. [86]
2020	Diverse	*Arundo donax* and *Phragmites australis* (Poaceae)	Phytoliths in reed shoots	Delplace et al. [87]
2020	Cadmium (Cd)	*Urochloa decumbens*, *Urochloa brizantha*, and *Megathyrsus maximus* (Poaceae)	Phytoliths in grass shoots	de Melo Farnezi et al. [88]
2021	Arsenic (As)	*Phragmites japonicus* (Poaceae) and *Thelypteris palustris* (Thelypteridaceae)	Phytoliths in plant shoots	Min et al. [89]
2022	Diverse	*Triticum aestivum* (Poaceae)	Phytoliths in inflorescences, leaf sheaths, and stems	Liu et al. [90]
2023	Diverse	*Triticum aestivum* (Poaceae)	Phytoliths in leaf sheaths, stems, and panicles	Liu et al. [91]

**Table 2:** Examples of metal(loid) accumulating terrestrial plants and information on corresponding Si contents.

Metal(loid)	Plant Species	Plant Family	Si Content ^4^ (%)
Arsenic (As)	*Pteris vittata* ^1^	Pteridaceae	ns
	*Pteridium aquilinum* ^2^	Dennstaedtiaceae	1.5
	*Corrigiola telephiifolia* ^2^	Caryophyllaceae	ns
	*Sacciolepis cymbiandra* ^2^	Poaceae	ns
Cadmium (Cd)	*Arabidopsis halleri* ^1^	Brassicaceae	ns
	*Malva rotundifolia* ^2^	Malvaceae	ns
	*Abelmoschus manihot* ^2^	Malvaceae	ns
	*Pterocypsela laciniata* ^2^	Asteraceae	ns
	*Lantana camara* ^2^	Verbenaceae	ns
Chromium (Cr)	*Brachiaria mutica* ^2^	Poaceae	ns
	*Leptochloa fusca* ^2^	Poaceae	ns
	*Canna indica* ^2^	Cannaceae	0.4
Cobalt (Co)	*Haumaniastrum robertii* ^1^	Lamiaceae	ns
Copper (Cu)	*Aeolanthus biformifolius* ^1^	Lamiaceae	ns
	*Brassica campestris* ^2^	Brassicaceae	ns
	*Helianthus annuus* ^2^	Asteraceae	0.03
Lead (Pb)	*Noccaea rotondifolia* subsp. *cepaeifolia* ^1^	Brassicaceae	ns
	*Pinus sylvestris* ^2^	Pinaceae	0.2
	*Quercus robur* ^2^	Fagaceae	0.6
Mercury (Hg)	*Plectranthus* sp. ^2^	Lamiaceae	0.07 (*P. japonicus*)
	*Clidemia* sp. ^2^	Melastomataceae	ns
	*Capsicum annuum* ^2^	Solanaceae	0.05
	*Phyllanthus niruri* ^2^	Phyllanthaceae	ns
	*Inga edulis* ^2^	Fabaceae	ns
Nickel (Ni)	*Berkheya coddii* ^1^	Asteraceae	ns
	*Brassica juncea* ^2^	Brassicaceae	ns
	*Typha angustifolia* ^2^	Typhaceae	0.02
Tin (Sn)	*Cyperus rotundus* ^3^	Cyperaceae	ns
	*Imperata cylindrica* ^3^	Poaceae	0.6
Zinc (Zn)	*Noccaea caerulescens* ^1^	Brassicaceae	ns
	*Sinapis arvensis* ^2^	Brassicaceae	ns
	*Tagetes erecta* ^2^	Asteraceae	ns

^1^ taken from Reeves et al. [23], ^2^ taken from Sharma et al. [19], ^3^ taken from Ashraf et al. [102], ^4^ taken from Hodson et al. [26], ns = not specified.

## Data Availability

All relevant data are presented within the paper.
